# Decoding Tocopherol–Polyphenol interactions in oil-in-water emulsions through combined WIM-CAT and CV assays

**DOI:** 10.1016/j.crfs.2026.101344

**Published:** 2026-02-09

**Authors:** Camille Robichon, Erwann Durand, Jayaruwan G. Gamaethiralalage, Philippe Bohuon, Bruno Baréa, Nathalie Barouh, Francis Courtois, Frédéric Fine, Louis C.P.M. de Smet, Pierre Villeneuve

**Affiliations:** a CIRAD, UMR QualiSud, Montpellier, F-34398, France; b QUALISUD, Univ Montpellier, Avignon Université, CIRAD, Institut Agro, IRD, Université de La Réunion, Montpellier, France; c Wageningen University & Research, Laboratory of Organic Chemistry, 4 Stippeneng, Wageningen, 6708 WE, the Netherlands; d TERRES INOVIA, Parc Industriel – 11 Rue Monge, Pessac, 33600, France

**Keywords:** Tocopherols, Curcumin, Quercetin, Synergy, Modelling, Cyclic voltammetry, Antioxidants

## Abstract

The antioxidant interactions of α- and γ-tocopherol with curcumin and quercetin were assessed in an oil-in-water emulsion using the WIM-CAT assay, a method integrating Weibull interaction modeling with the conjugated autoxidizable triene technique. Synergistic effects were strongest for γ-tocopherol with curcumin and for α-tocopherol with quercetin, particularly at low tocopherol concentrations (0.2 μM in emulsion, 380 ppm in oil) and high molar ratios (3:1). Increasing tocopherol concentration to 0.6 μM in emulsion (1140 ppm in oil) reduced synergy, likely reflecting pro-oxidant activity. The presence of ferrous ions accelerated oxidation but did not influence synergistic interactions, while acidic conditions reduced tocopherol pro-oxidation and modified the effects of curcumin and quercetin. Weibull modeling revealed isoform-dependent differences during the propagation phase of oxidation. Cyclic voltammetry further suggested that the synergy of α-tocopherol may involve antioxidant regeneration mechanisms, whereas γ-tocopherol appears to act through alternative redox processes. Together, kinetic and electrochemical analyses provide complementary insights into the conditions governing antioxidant interactions.

## Highlights

• WIM-CAT and voltammetry reveal isoform-specific antioxidant synergy in emulsions.
• γ-Tocopherol–curcumin and α-tocopherol–quercetin show highest synergy at low TOH.
• High tocopherol levels induce pro-oxidant effects and reduce antioxidant synergy.
• Weibull modeling distinguishes polyphenol effects on initiation and propagation.
• pH and Fe^2+^ strongly modulate antioxidant efficiency in PUFA-rich emulsions.

## Graphical abstract

**Figure:**
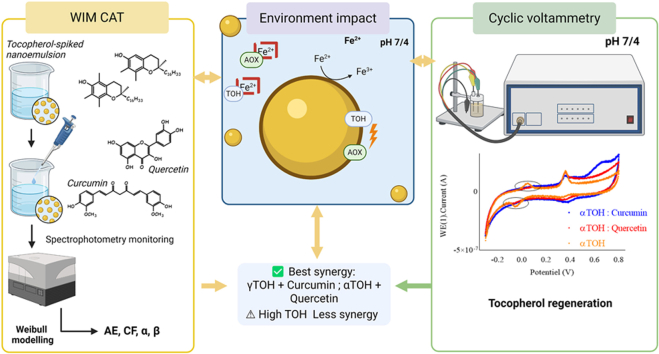

## 1 Introduction

In recent years, the European Food Safety Authority (EFSA) has advised increasing the intake of polyunsaturated fatty acids (PUFAs) due to their recognised health benefits (Dael, 2021). However, a major challenge associated with PUFA-enriched products is their limited oxidative stability, which significantly shortens shelf life. Due to their chemical structure, PUFAs are highly susceptible to lipid oxidation, which deteriorates their sensory and nutritional qualities and poses health risks. Notably, the consumption of oxidized lipids has been associated with an increased risk of cardiovascular diseases (Gianazza, 2019; Schaich, 2024). At the same time, consumer preferences have shifted toward clean-label products that feature fewer ingredients, a greater use of natural rather than synthetic compounds, enhanced nutritional profiles, and closer alignment with global health recommendations. This trend has driven the continuous growth of the natural antioxidants market, highlighting the need for effective solutions to counteract lipid oxidation (Ghosh et al., 2022). Among natural antioxidants, tocopherols (TOH), ascorbic acid and extracts from botanical plants are widely used. TOH are abundant in most vegetable oils and other plant species; and different TOH isoforms (α, β, γ, δ) exist depending on the position and the number of methyl groups on the polar chromanol head. These structural differences influence antioxidant efficiency by affecting bond energies and hydrogen-donating ability, which are correlated with the capacity to form stable resonance (mesomeric) structures (Barouh et al., 2022). Moreover, variations in the polarity of the chromanol structure can affect the distribution of TOH within the food matrix, thereby modulating their antioxidant activity (Wang et al., 2022). Research on emulsions has grown substantially in recent decades, as many formulated foods themselves are emulsions. In particular, lipid-containing foods are often heterogeneous systems where lipids are dispersed in an aqueous phase. These structures therefore provide highly relevant models for studying the complex organization and behavior of modern formulated foods (Berton-Carabin et al., 2014). In emulsions, the behavior of TOH remains complex. For instance, the accumulation of oxidized TOH derivatives enhanced by higher concentrations or the presence of prooxidant metal ions can shift their role from antioxidant to prooxidant activity, thereby compromising product stability (Barouh et al., 2022). To mitigate this phenomenon, strategies based on antioxidant synergy are proposed. Such synergistic interactions encompass a wide range of mechanisms that can extend antioxidant protection and limit their conversion to a prooxidant form. One promising approach involves combining TOH with other antioxidants to create synergistic effects, either by complementary radical scavenging and metal chelating activities, as mentioned by Bayram and Decker (2023) (Bayram and Decker, 2023) or by improving the spatial distribution of antioxidants within the food matrix, a critical parameter in controlling lipid oxidation as described in the case of phospholipid synergy by Tang et al. (2023) (Tang et al., 2023). Within this broader framework of synergy, regeneration of TOH represents a specific mechanism that further contributes to maintaining their protective effects (Barouh et al., 2022). Curcumin emerges as a possible candidate for such synergistic combinations with TOH. It is the most widely used polyphenol as a food additive and is well-documented for its antioxidant, anti-inflammatory, and antitumor activities (Kharat et al., 2017; Le Bloch et al., 2024). Similarly, quercetin, a well-known flavonoid, has been reported to act synergistically with TOH, enhancing overall antioxidant performance (Becker et al., 2007, 2024). However, multiple factors can influence antioxidant efficiency and synergy in food emulsions, highlighting the need to contextualize results in lipid oxidation studies. For instance, characteristics of the aqueous phase, such as pH or the presence of metal ions, can significantly influence the behavior and synergistic interactions of antioxidants (Bayram et al., 2023). Acidic pH or ferrous ion contamination during processing must be carefully considered to accurately mimic the behavior of food products. One way to assess the influence of pH and pro-oxidant metal ions is through model systems that enable evaluation of antioxidant combinations. In that context, the recently developed WIM-CAT assay (Robichon et al., 2025), based on tung oil oxidation, is a rapid and tuneable method for assessing antioxidant activities in oil-in-water emulsions. This oil, although not being a classical food grade oil, is very convenient to follow lipid oxidation and evaluate antioxidant efficacies. Moreover, WIM-CAT assay combines the results with mathematical analysis to better evaluate the impact of the composition (pH, presence of pro-oxidant metals, buffer, etc) on the antioxidant interaction (synergistic or not) in the oil-in-water emulsion. In the present study, the WIM-CAT assay evaluates the ability of curcumin and quercetin to act synergistically with α- and γ-TOH, as well as how the composition of the emulsion and the choice of TOH isoforms influence these interactions. Additional environmental factors, such as pH or metal ion presence, are considered to further understand their impact on antioxidant synergy. While the WIM-CAT model enables highlighting the conditions for the synergistic effects and hypotheses behind them, it does not identify which specific mechanisms are involved. Among these mechanisms, TOH regeneration is one possible process contributing to the maintenance of antioxidant protection and the prevention of TOH conversion to a prooxidant form. In the second part, cyclic voltammetry (CV) was used to perform an in-depth analysis of electron transfer, providing further insights into redox reactions and helping to assess potential thermodynamic TOH regeneration, as well as the impact of environmental factors such as pH.

## 2 Materials and methods

### 2.1 Chemicals

Tung oil from *Aleurites for*dii seeds (tung oil, average MW = 872 g/mol, Iron content <0.9 mg/kg), curcumin (78 %), quercetin (≥98%), αTOH (≥97 %), γTOH (≥98 %), TOH standards, polyoxyethylene(23)lauryl ether (Brij 35, estimated MW = 1199 g/mol), iron chloride (II) were all purchased from Sigma (Saint Quentin, France). All used solvents were HPLC or analytical grades and were purchased from Sigma (Saint Quentin, France).

### 2.2 Measurement of peroxide value

Peroxide value (PV) measurement in tung oil was made by iodometric titration according to the international norm ISO 3960. Typically, a known quantity of oil (0.5–2 g) was dissolved in chloroform (10 mL) in a 50 mL round-bottom flask, and acetic acid (15 mL) was added. To initiate the reaction, a freshly prepared saturated potassium iodide (1 mL) was added. The flask was then capped and stirred (1 min). After 5 min in the dark at room temperature, the reaction was stopped by adding distilled water (75 mL). The manual titration was made with a sodium thiosulfate solution (0.002 N) with iodine as an indicator, and the PV was determined as follows:

(1) $$PV=\frac{\left(V-V0\right)\times N\times 1000}{m}$$

With *V*, the volume (mL) of the sodium thiosulfate solution in the presence of the sample. *V*0 is the volume (mL) of the sodium thiosulfate solution in the absence of the sample, *m* is the mass of the sample (g), and *N* means the normality of the sodium thiosulfate solution. The results obtained using this method were expressed in meqO_2_/Kg of oil.

### 2.3 Stripping of tung oil

Tung oil was stripped from its polar compounds (including TOH) by passing 25 mL of a hexane solution of tung oil (500 mg/mL) in a Redisep column prefilled with alumina (Cat: 19.997-4, 50g), connected to a Reveleris X2 flash chromatography system (Buchi, Villebon sur Yvette, France). The solution was eluted with hexane, and the fractions were selected according to HPLC analysis. After complete removal of TOH, verified by HPLC (see section Quantification of TOH by HPLC-fluorescence). The obtained stripped tung oil solution can be stored in the dark at 4 °C for up to 1 day before use. Then, the hexane was evaporated under vacuum (∼20 kPa) at 30 °C using a rotary evaporator equipped with a vacuum pump (Laborport, KIF Neuberger GmbH, Freiburg, Germany). Finally, the stripped tung oil was aliquoted into brown glass tube (4 mL), then the solvent residues were removed by bubbling with a stream of nitrogen.

### 2.4 Determination of Total Fatty Acid (TFA) profiles of tung oil

The TFA profiles of tung oil were determined by gas chromatography (GC) according to the NF T60-233 standard method with slight modifications. In a 15 mL tube, sodium methylate solution (1 mL) with phenolphthalein was added to oil sample (15 mg). After heating at 65 °C for 10 min, hydrochloric methanol (1 mL) was added. The mixture was again heated at 65 °C for 10 min. After cooling, hexane (1 mL) and water (1 mL) were added. The mixture was centrifuged at 1500 rpm for 5 min using a Hettich Rotina 380R (Hettich, Tuttlingen, Germany), and the organic phase was collected. A 1 μL aliquot of the organic layer was then injected into the GC system. GC analyzes were performed with a Focus GC (Thermo Electron Corporation, Massachusetts, USA), equipped with a split injector (ratio of 1/20), CP-Cil 88 Varian capillary column (50 m × 0.25 mm with 0.2 μm film thickness; Agilent Chrompack), and helium (1 mL min^−1^) as carrier gas. Fatty acid methyl esters (FAME) were analyzed by a flame ionization detector and ChromCard software data system (version 2005, Thermo FisherScientific, Massachusetts, USA). The column temperature started from 150 °C, reached 225 °C with a rise of 5 °C.min^−1^ and was maintained at 225 °C for 10 min. The injector and detector temperatures were 250 and 270 °C, respectively. FAME were identified using the retention time of external standards of FAME mixture (mix37 EMAG Supelco). (C16:0, 1.9%; C18:0, 2.1%; C18:1n9c, 4.9%; C18:1n7, 0.2%; C18:2n6c, 6.7%; C20:1, 0.7%; C18:3 (α-eleostearic acid), 79.8%; C18:3 (β-eleostearic acid), 3.6%)

### 2.5 Quantification of tocopherols (TOH) by HPLC-fluorescence

The amounts of TOH in tung oil were evaluated using oil samples (10 mg) diluted in hexane (10 mg/mL). Four distinct TOH (α, β, γ and δ) were quantified according to the ISO-FDIS 9936 standard. HPLC analysis was performed using an Ultimate 3000 with a fluorescence detector FL 3000 (Thermo electron, Massachusetts, USA) equipped with an ACE silica column 250 mm × 4.6 mm, 5 μm (A.I.T. France, Corneilles en Parisis, France). The mobile phase consisted of hexane/1,4 dioxane (97:3 v/v) with a flow rate was 1.3 mL min^−1^. The column temperature was maintained at 27 °C. Fluorescence detection was set at 290 and 330 nm for excitation and emission, respectively. The injection volume was 100 μL and the calibration curves were realized with standard solutions of each TOH’ isoform (reference 613424).

### 2.6 Incorporation of TOH in tung oil

An ethanol solution of TOH was prepared at approximately 1.21 g.L^−1^. This solution (1 mL) was then evaporated under nitrogen, and stripped tung oil (800 μL) was added. The obtained oil sample loaded in TOH was then diluted with stripped tung oil to give two TOH concentrations set at 1.21 g.L^−1^ (1140 ppm) and 0.4 g.L^−1^ (380 ppm), respectively. The concentration was subsequently confirmed by HPLC (see Section 2.5).

### 2.7 Preparation of tung oil-in-water nanoemulsions

In a 250-mL Erlenmeyer flask, a Brij 35 solution (150 mL, 19.8 mg.L^−1^) and tung oil (32 μL) (with or without TOH) were incorporated and roughly vortexed for 30 s, then passed through a Silverson L5M-A (Silverson, Silverson France) at 10,000 rpm for 3 min. The second antioxidant (AOX) was added post-emulsification. Incorporation was carried out using ethanol solutions of the antioxidants, the samples were vortexed for 30 s without an intermediate stabilisation phase. The concentration of TOH was set at 0.2 μM (T0.2) and 0.6 μM (T0.6) in the nanoemulsion, corresponding to 380 ppm and 1140 ppm, respectively, in the tung oil. The molar ratios of TOH to the second antioxidant were set at 1:3 (3x), 1:1 (1x) and 1:0.3 (0.3x).

### 2.8 Monitoring oxidation in tung oil-in-water nanoemulsion

The 96-well microplate (Greiner) was filled with nanoemulsion (115 μL) and brought to a final volume of 250 μL with water. In the case of ferrous ion initiation, a 2.5 μM FeCl_2_ solution (10 μL) was added and completed with an aqueous solution to obtain 250 μL. The microplate was placed in a wet chamber of the SPARK 10M TECAN reader (Tecan, Gröedig, Austria). An initial absorbance measurement was recorded at λ = 234 nm to assess the presence of conjugated dienes and to verify the state of oxidation of the oil at the start of the kinetic process. Absorbance values (λ = 273 nm) were recorded every 10 min for the first 2 h, then every hour until all the samples have reached a threshold signifying the end of oxidation.

### 2.9 Particle size measurement using Dynamic Light Scattering (DLS)

The nanoemulsion droplet size distribution was determined by laser light scattering using a Zetasizer pro (Malvern Panalytical, France) equipped with ZS Xplorer program (Malvern Panalytical, France). Measurements were performed at 25 °C in fresh nanoemulsions (day 0) and at the end of the storage period (day 4), to evaluate oil droplet stability. Nanoemulsion samples were suitably ten times diluted in distilled water (pre-filtered through a 0.2 μm RC filter), and the refractive index of tung oil and water at 25 °C was taken as 1.47 and 1.33, respectively. Measurements were carried out in triplicate and results were given in terms of Z-average (μm) and polydispersity index (PI). At day 0, the average droplet size was 196.5 ± 16.8 nm (PDI = 0.3). By day 4, the droplet size remained stable at 182.3 ± 7.3 nm (PDI = 0.3), confirming the long-term stability of the nanoemulsion throughout the study.

### 2.10 Mathematical modeling

The WIM CAT Assay was carried out according to our previous work (Robichon et al., 2025), in which the kinetic model describing lipid oxidation in nanoemulsion systems is detailed. For full methodological details and a full justification of the model structure, the reader is referred to our previous publication. Briefly, the model is based on the reduced absorbance $A^{*}$, defined as:

(2) $$A^{*}=\frac{A-A_{\infty }}{A_{0}-A_{\infty }}$$

where A is the absorbance at time t, A_0_ the initial absorbance at t = 0, and A∞ the final absorbance after complete oxidation. Beyond a characteristic induction period, denoted *LagP*, the evolution of $A^{*}$ over time is described by a Weibull-type distribution:

(3) $$A^{*}=\left\{ \;\begin{array}{c} 1,\text{for}\;t\leq LagP\\ \exp\left[-{\left(\frac{t}{\alpha }\right)}^{\beta }\right],\text{for}\;t\;>\;LagP \end{array}\right.$$

Here, *LagP* corresponds to the duration of the lag phase, during which the antioxidant delay the onset of radical chain reactions and thereby reduce the oxidation rate. The parameters *α* and *β* are the scale (a characteristic time) and the shape parameters of the degradation curve, respectively. The first-order model is a special case of Eq. (3), where β=1. This approach accounts for the heterogeneity in oxidation dynamics due to droplet size distribution and oxidative resistance, inherent to emulsification processes. The probability density function (PDF) corresponding to the model was derived by differentiating Eq. (3) with respect to time, yielding:

(4) $$\frac{dA^{*}}{dt}=-\frac{\beta }{\alpha }{\times \left(\frac{t}{\alpha }\;\right)}^{\beta }\times \exp\;\left[-{\left(\frac{t}{\alpha }\;\right)}^{\beta }\;\right]=-\text{PDF}$$

### 2.11 Kinetic parameter estimation

The Weibull parameters (*α* and *β*) were determined using the Curve Fitting Toolbox 3.6 in MATLAB® (The MathWorks Inc., Natick, MA, USA) by applying the Nonlinear Least-Squares method. The goodness of fit was assessed by the root mean square error (RMSE) of the reduced absorbance and the adjusted R^2^. Confidence intervals for both parameters (IC_*α*_ and IC_*β*_) were calculated at the 95 % confidence level. Within this model, the *LagP* was defined as the time at which the probability density function (PDF) (4) of the model reaches a threshold value of 5 × 10^−3^ h^−1^. The confidence interval for *LagP* was computed by propagating the uncertainties in *α* and *β* using a Monte Carlo simulation with 10,000 iterations.

### 2.12 Cyclovoltammetry

Cyclic voltammetry (CV) cycles were recorded using a glassy carbon working electrode (Metrohm BV, The Netherlands), a platinum counter electrode (Metrohm), and an Ag/AgCl (3 M KCl) reference electrode (Metrohm), connected to a Metrohm Autolab PGSTAT302N potentiostat. Before each experiment, the glassy carbon electrode was carefully polished using a 0.3 μm alumina slurry on a polishing pad, rinsed thoroughly with distilled water, and dried. Working solutions consisted of 0.1 mol.L^−1^ NH_4_PF_6_ (Sigma, Lot #MKCV8722, CAS 16941-11-0, purity 99.98%) in a 50% ethanolic solution. The pH of the solution was adjusted using a 1 M NaOH solution. The electrode was first cycled in the supporting electrolyte until a stable baseline was achieved. Subsequently, the analyte was introduced into the cell, and CV were recorded. All potentials reported herein are referenced to the Ag/AgCl (3 M KCl) electrode.

### 2.13 Statistical analysis

Each kinetic was carried out in triplicate. The results were expressed as the mean of the triplicates (mean values ± standard deviations). Different tests were performed according to the relationship between samples and group characteristics to determine which mean values were different (significance level was set at p < 0.05): One-way ANOVA calculations included sum of squares (SSB, SSW) and F statistic, with Tukey's HSD post-hoc test. Paired data were analyzed using a paired *t*-test. Independent groups were analyzed using an independent *t*-test. Statistical analyzes were performed in MATLAB using user-defined means, standard deviations and sample sizes to simulate the data.

## 3 Results and discussion

An effective strategy to enhance the antioxidant activity of tocopherols is to exploit synergistic interactions with other antioxidants. Such synergism may arise from complementary mechanisms, including the combination of radical-scavenging and metal-chelating activities (Bayram and Decker, 2023), or from improved localization of antioxidants within the food matrix (Tang et al., 2023). Within this framework, the regeneration of tocopherols (TOH) represents a distinct synergistic mechanism that contributes to maintaining their protective function (Barouh et al., 2022). Among potential synergistic partners, curcumin and quercetin stand out as strong candidates. For instance, Zhang et al. (2023) reported that quercetin exhibited the strongest synergistic effect with α-tocopherol (αTOH) at a 2:1 ratio in an olive oil model system. Similarly, Pedrielli and Skibsted (2002) described a synergistic antioxidant effect between quercetin and αTOH in homogeneous methyl linoleate systems. Synergism between curcumin and αTOH has also been reported when both compounds were co-encapsulated in bicosomes for pharmaceutical applications (Vergara et al., 2023). In contrast, other studies did not observe any synergistic effect between curcumin and αTOH when this latter was loaded into micelles (Dai et al., 2009). In this context, we further investigated in the present study the potential synergistic effects of quercetin and curcumin in combination with either α-tocopherol or γ-tocopherol.

### 3.1 Tocopherols interaction with AOX at neutral pH (pH 7)

The kinetic measurements were performed in triplicate for each experimental condition, and the resulting datasets were fitted using the Weibull kinetic model. The model showed good performance across all conditions, as reflected by low root mean square error (RMSE) values and high coefficients of determination (R^2^), consistent with previsoulsy reported findings (Robichon et al., 2025) (Table S1, Supplementary Data). This modeling approach enabled the precise determination of the *LagP* by evaluating the first derivative (see Section 2.10). Once the *LagP* values for individual antioxidants (TOH and AOX) and their combinations (TOH–AOX) were obtained, the combination factor (*CF*) was calculated to quantify the nature of their interaction as follows:

$$CF=\frac{LagP\left(TOH-AOX\right)}{\left[LagP\left(TOH\right)+LagP\left(AOX\right)\right]}$$

*CF* > 1 implies synergy, *CF* < 1 indicates antagonism, and *CF* = 1 reflects an additive effect. While related, *CF* differs from antioxidant efficiency (*AE*), which refers to the absolute *LagP* value of each antioxidant or their combination. Evaluating antioxidant combinations thus requires considering both *CF* and *AE*. When *CF* is plotted against *AE* (based on *LagP*), distinct patterns emerge that highlight the effectiveness of the combination, as described by Robichon et al. (2025) Ultimately, *CF* arises from the balance between the physicochemical reactivities of antioxidants and the prooxidant mechanisms, which may act independently or synergistically.

**Fig. 1:**
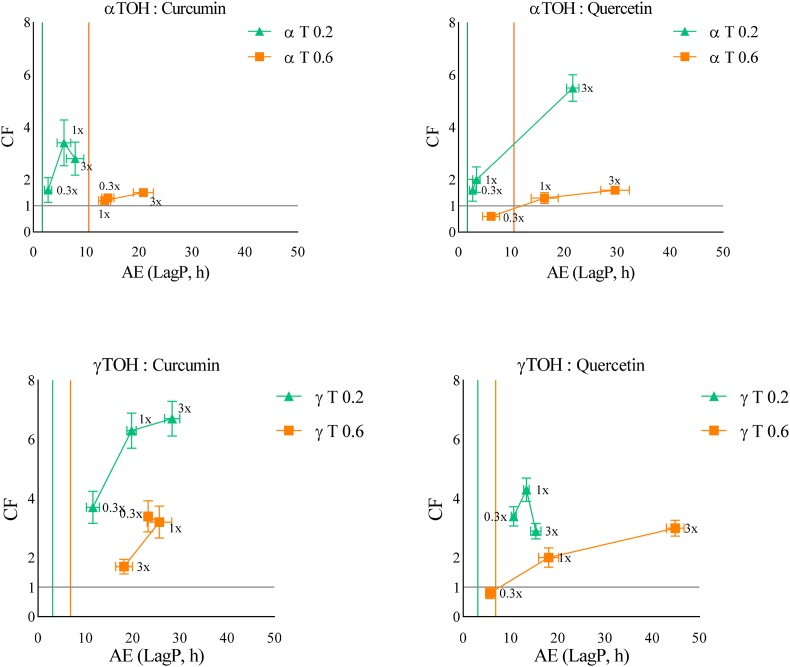
Effect of increasing αTOH or γTOH to AOX ratios (0.3x (1:0.3), 1x (1:1), 3x (1:3)) on the CF and AE using two TOH concentrations (0.2 and 0.6 μM in emulsion) at neutral pH. (Green Triangles = αTOH or γTOH at 0.2 μM in emulsion. Orange squares = αTOH or γTOH at 0.6 μM in emulsion). Coloured vertical lines represent the LagP of TOH at each specific concentration. The horizontal line represents the separation between an antagonistic combination (below, CF < 1) and a synergistic combination (above, CF > 1).

In these experiments, the molar ratios of TOH to the second antioxidant (curcumin or quercetin) were set at 1:3 (3x), 1:1 (1x), and 1:0.3 (0.3x), allowing the assessment of synergistic effects across different relative concentrations. As observed in Fig. 1, the combinations were generally synergistic. Maximum *CF* values were observed with γTOH combined with curcumin, and with αTOH combined with quercetin, both at T0.2 and a 3x molar ratio. However, at higher concentrations of TOH (T0.6), *CF* values were generally lower, with the highest value obtained with γTOH combined with curcumin both at 0.3x and 1x ratios, or with quercetin at a 3x ratio. This may be attributed to the pro-oxidant behavior of TOH when used in elevated amounts, which can reduce their *AE* and result in a shorter-than-expected *LagP*. This effect was less pronounced when γTOH was combined with quercetin, as the difference in *CF* values was smaller compared to the other conditions.

If we focus on curcumin, it exhibited predominantly synergistic interactions with TOH, with a stronger effect observed with γTOH than with αTOH, reaching a *LagP* of nearly 30 h at a 3x molar ratio at γT0.2. This difference is hypothesized to result from the distinct partitioning behaviors of the TOH isoforms, as mentioned by Wang et al. (2022) (Wang et al., 2022), in which γTOH is located more at the interface compared to αTOH, potentially influencing its capacity to interact with a second antioxidant such as curcumin. However, two distinct patterns emerged depending on either TOH were added at low or high concentration. Indeed, at lower concentration (T0.2), for both γTOH and αTOH, a maximum *CF* value were reached at 1x ratio. On possible explanation result in the accumulation of oxidized products, which can hinder interactions between antioxidants. Another reason is the enolic form of curcumin, which can dimerize at high concentrations. While these dimers may still exhibit antioxidant activity, they may be less able to interact effectively with TOH under such conditions (Masuda et al., 2002). At higher TOH levels (T0.6), distinct isoform-dependent behaviors were observed, indicating that additional mechanisms may contribute to the response. For γTOH, a declining trend was observed: *AE* slightly decreased as curcumin concentration increased, with the lowest *LagP* recorded at a 3x ratio. This effect may reflect an unfavorable balance between pro-oxidant and antioxidant mechanisms, resulting in a modified yet still synergistic response. For αTOH, only the highest curcumin concentration produced a notable increase in *LagP*. The potential pro-oxidant behavior of αTOH at elevated concentrations may disrupt its synergy with other antioxidants, thereby limiting the overall *AE* (Barouh et al., 2022). However, in light of our overall observations with curcumin at neutral pH, it should be noted that curcumin is known to be chemically unstable under these pH conditions. Therefore, part of the apparent consumption of curcumin in the kinetic model may result from its autohydrolysis/degradation, independent of the lipid oxidation reaction.

Regarding quercetin, it exhibited overall synergistic interactions with TOH, with a stronger effect observed in combination with αTOH than with γTOH at low concentration (T0.2). With αTOH, *LagP* values reached approximately 30 h, while with γTOH *LagP* extended to nearly 50 h, highlighting the strong antioxidant potential of quercetin under these conditions. These differences may reflect the distinct partitioning of TOH isoforms within emulsions, as well as the combined contribution of distribution and regeneration effects (Wang et al., 2016). Increasing quercetin concentrations generally resulted in longer *LagP* and higher *CF* values, suggesting that a favorable distribution of antioxidants within the matrix may enhance their interactions, possibly through regeneration mechanisms. A different behavior is observed at γT0.2, where a maximum *CF* is reached at 1x molar ratio.

### 3.2 Tocopherols interaction with AOX at acidic pH (pH 4)

**Fig. 2:**
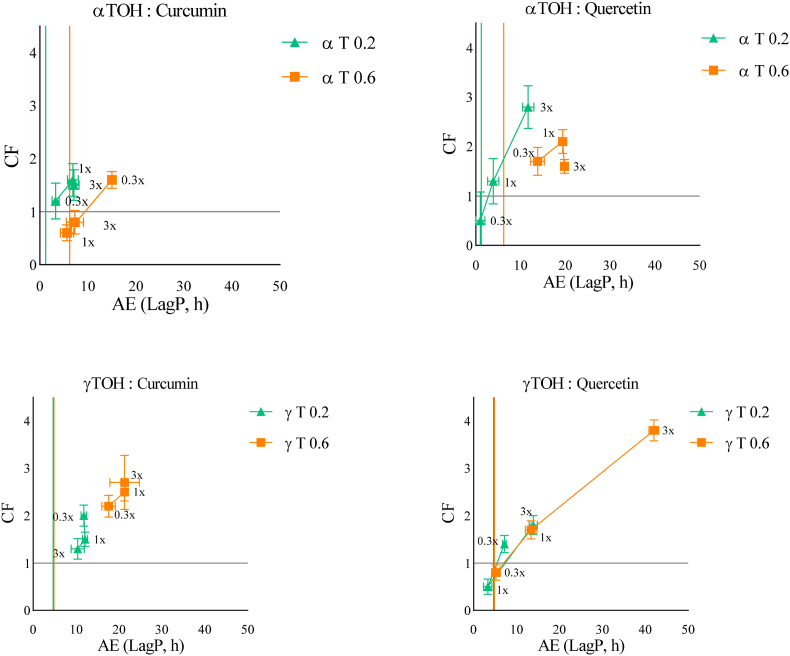
Effect of increasing αTOH or γTOH to AOX ratios (0.3x (1:0.3), 1x (1:1), 3x (1:3)) on the CF and AE using two TOH concentrations (0.2 and 0.6 μM in emulsion) at acidic pH (pH 4). (Green Triangles = αTOH or γTOH at 0.2 μM in emulsion. Orange squares = αTOH or γTOH at 0.6 μM in emulsion). Coloured vertical lines represent the LagP of TOH at each specific concentration. The horizontal line represents the separation between an antagonistic combination (below, CF < 1) and a synergistic combination (above, CF > 1).

Lowering the pH to acidic conditions (pH 4) significantly altered the trends previously observed (Fig. 2). The highest *CF* was observed for γT0.6 in combination with quercetin or curcumin at a 3x molar ratio. The influence of their concentrations differed from that observed at neutral pH. Under acidic conditions, *CF* values were comparable at T0.2 and T0.6, suggesting a reduced pro-oxidant behavior of TOH when added at high concentration, compared to neutral pH. This effect may be related to the greater stability and lower reactivity of αTOH oxidation products, such as tocopherone cation (TO^+^). The combination with curcumin appeared to be more affected than that with quercetin, likely due to curcumin's sensitivity to low pH. Indeed, under acidic conditions, curcumin predominantly adopts its keto form, which favors self-assembly into spherical curcumin nanoparticles (CNPs) and increases aggregation (Kaur et al., 2018). This aggregation reduces the effective availability of curcumin for interaction with tocopherols and may therefore limit hydrogen-donation reactions and overall antioxidant efficiency.

### 3.3 Impact of the presence of added ferrous ions on the different antioxidant combinations

**Fig. 3:**
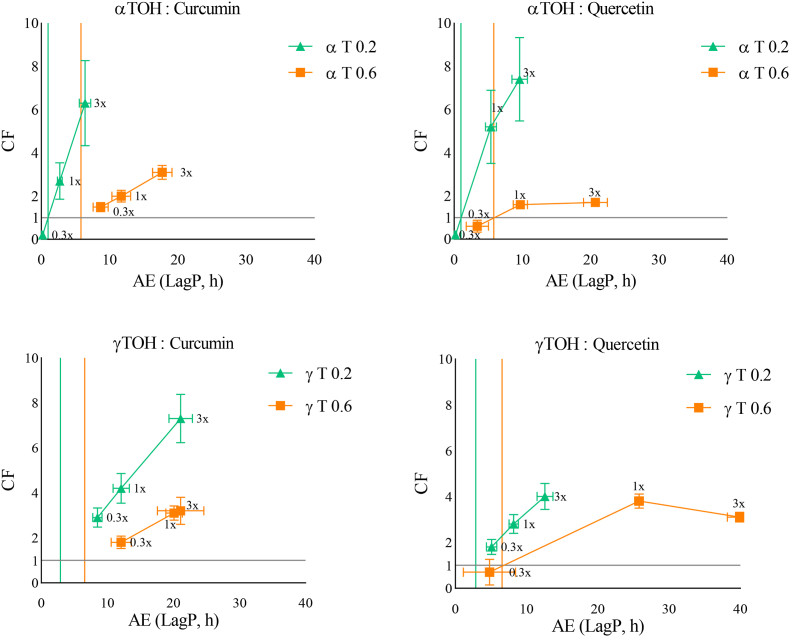
Effect of increasing αTOH or γTOH to AOX ratios (0.3x (1:0.3), 1x (1:1), 3x (1:3)) on the CF and AE using two TOH concentrations (0.2 and 0.6 μM in emulsion) at neutral pH, in the presence of ferrous ions (0.1 μM). (Green Triangles = αTOH or γTOH at 0.2 μM in emulsion. Orange squares = αTOH or γTOH at 0.6 μM in emulsion). Coloured vertical lines represent the LagP of TOH at each specific concentration. The horizontal line represents the separation between an antagonistic combination (below, CF < 1) and a synergistic combination (above, CF > 1).

**Fig. 4:**
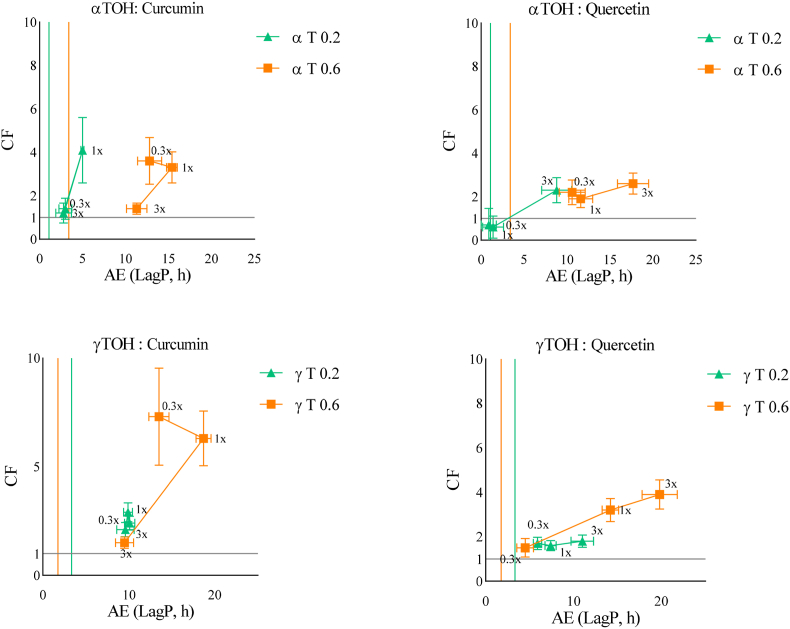
Effect of increasing αTOH or γTOH to AOX ratios (0.3x (1:0.3), 1x (1:1), 3x (1:3)) on the CF and AE using two TOH concentrations (0.2 and 0.6 μM in emulsion) at acidic pH (pH 4), in the presence of ferrous ions (0.1 μM). (Green Triangles = αTOH or γTOH at 0.2 μM in emulsion. Orange squares = αTOH or γTOH at 0.6 μM in emulsion). Coloured vertical lines represent the LagP of TOH at each specific concentration. The horizontal line represents the separation between an antagonistic combination (below, CF < 1) and a synergistic combination (above, CF > 1).

The same experiments were conducted in the presence of ferrous ions (0.1 μM) at neutral (Fig. 3) and acidic pH (Fig. 4). This ferrous ion concentration was selected to fall within the range typically found in conventional food-grade refined oils. As expected, Fe^2+^ significantly accelerated oxidation kinetics at neutral pH, leading to a substantial reduction in *LagP*. The lowest *LagP* occurred with αT0.2 combined with a 0.3x molar ratio of quercetin or curcumin, whereas the highest *LagP* was obtained with γT0.6 and quercetin at a 3x molar ratio. A linear concentration-dependent effect was observed for both curcumin and quercetin, with the highest *CF* values obtained with αT0.2 in combination with curcumin. These results seem to support the hypothesis of a combined effect, with both radical-scavenging and metal-chelating contributions of TOH and curcumin, or quercetin. Ak and Gülçin (2008) (Ak and Gülçin, 2008) reported curcumin's ability to chelate ferrous ions (Fe^2+^), while Roedig-Penman and Gordon (1998) (Roedig-Penman and Gordon, 1998) described a similar metal-chelating ability for quercetin.

Under acidic conditions (pH 4) and in the presence of ferrous ions (Fe^2+^), oxidation kinetics were not significantly accelerated (Fig. 4). Although higher *CF* were observed particularly for γT0.6 at a 1x molar ratio with curcumin and a 3x ratio with quercetin most combinations did not lead to a marked increase in *AE*. For curcumin, the effect appeared to depend on its concentration, possibly reflecting a dual mechanism: (i) the reduction of Fe^3+^ to Fe^2+^ may consume part of curcumin's reducing capacity, limiting tocopherol regeneration, and (ii) as under metal-free conditions, the predominance of the keto form and the formation of aggregates at acidic pH (Kaur et al., 2018) may further reduce its reactivity. In contrast, quercetin exhibited similar behavior to that observed without iron, though it appeared less affected by the presence of Fe^3+^, suggesting greater stability of its antioxidant function under these conditions.

3. Interpretation of Weibull parameters (*α* and *β*) for the analysis of the propagation phase of oxidation.

**Fig. 5:**
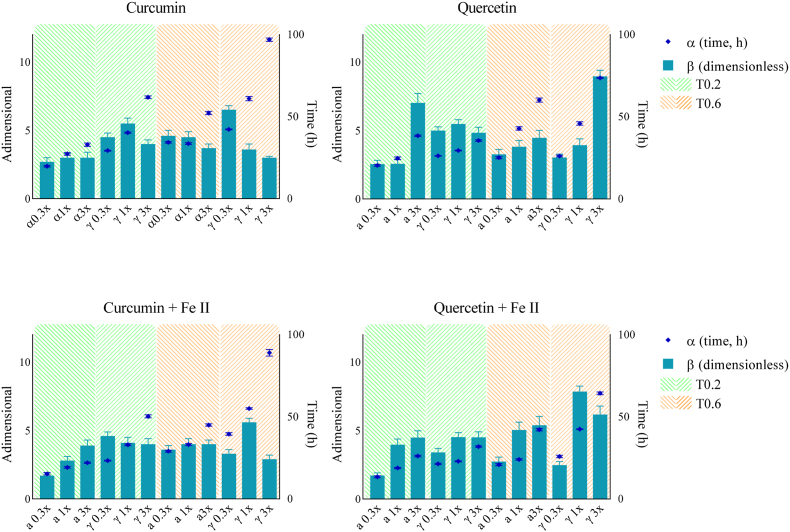
Effect of kinetic conditions on the evolution of the Weibull parameters α (scale) and β (shape) at neutral pH (pH = 7), in the absence (top) and presence (bottom) of ferrous iron addition.

**Fig. 6:**
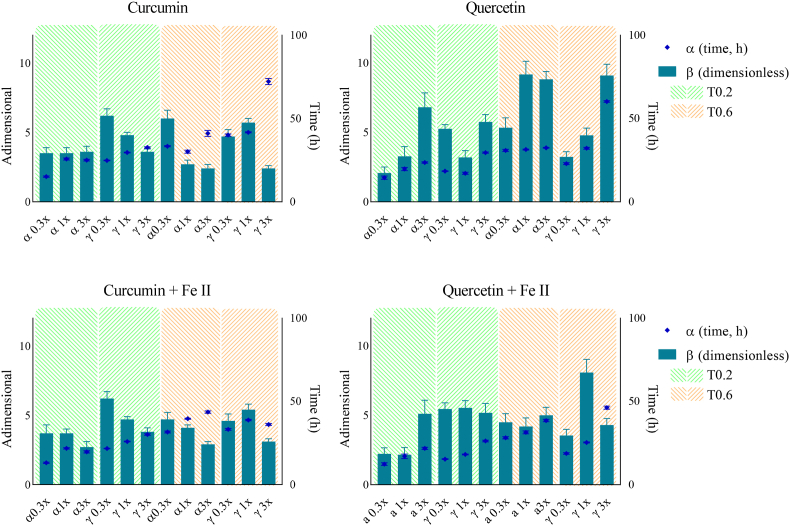
Effect of kinetic conditions on the evolution of the Weibull parameters α (scale) and β (shape) at acidic pH (pH = 4), in the absence (top) and presence (bottom) of ferrous iron addition.

Besides the *CF* and *AE* parameters, which are derived from *LagP* and primarily associated with the initiation phase of oxidation, the Weibull model provides complementary insights into the propagation phase. In this model, the scale parameter (α) represents the time at which substantial antioxidant degradation occurs, whereas the shape parameter (β) characterizes the kinetics of this degradation process. Practically, higher *α* values combined with lower β values generally indicate stronger antioxidant protection during the propagation phase. The *β* parameter is particularly informative, as it provides complementary information to *LagP* and helps elucidate how antioxidant composition influences oxidation behavior once the process enters its accelerated phase (Robichon et al., 2025). For instance, as shown in Fig. 5 (neutral pH) and Fig. 6 (acidic pH), increasing curcumin concentration consistently led to a decrease in β values. This trend persisted in the presence of ferrous ions under acidic conditions, suggesting that once oxidation enters the propagation phase, curcumin (and/or its oxidized derivatives) continues to exert a concentration-dependent protective effect. Thus, while αTOH exhibited pro-oxidant activity at high concentrations, accelerating oxidation initiation and shortening the lag phase, increasing curcumin concentration appeared to mitigate this negative effect during the propagation phase. In contrast, quercetin, which similarly increased *CF* and *AE* with concentration, produced an increase in β values, suggesting that once oxidation enters the propagation phase, quercetin (and/or its oxidized forms) may accelerate oxidation. This effect could arise from the pro-oxidant activity of its oxidation products, potentially enhancing the decomposition of primary oxidation intermediates (e.g., peroxides). Consequently, the synergistic antioxidant action of quercetin with TOH seems primarily restricted to the initiation phase, since at higher concentrations, pro-oxidant pathways may dominate during propagation.

The contradictory behaviors of TOH combinations with curcumin versus quercetin likely stem from variations in their respective oxidation by-products. It is well established that the antioxidant activity of certain polyphenols persists after oxidation, owing either to the intrinsic activity of their oxidized products or to subsequent reactions that generate new antioxidant species (Murakami et al., 2004; Hotta et al., 2002). This is the case for curcumin, whose dimeric oxidation product may account for up to 40% of its overall antioxidant efficiency, and for quercetin, whose oxidation products also retain antioxidant capacity (Masuda et al., 2002). However, the extent to which these by-products interact synergistically with TOH remains unclear. Furthermore, the β parameter revealed a clear distinction between the two tocopherol isoforms. Specifically, γTOH displayed higher β values than αTOH, particularly at neutral pH, indicating a faster degradation rate during the propagation phase. This difference was also observed under acidic conditions and in the presence of ferrous ions. Although the literature often reports that αTOH is preferentially consumed when multiple isoforms are present, the oxidation rates of the isoforms appear interdependent (Player et al., 2006). These findings underscore the potential influence of oxidized products (originating from both TOH and additional antioxidants) on the overall oxidation process, often overlooked in synergy analyses. Such products may exert either anti- or pro-oxidant effects, depending on their physicochemical properties (e.g., localization, interactions, redox potential) relative to their parent molecules. In conclusion, the WIM-CAT approach proves to be an effective tool for identifying antioxidant synergies and optimal interaction conditions, providing mechanistic insights into both the initiation and propagation phases of lipid oxidation. To further elucidate the underlying mechanisms, coupling this modeling approach with complementary experimental analyses would be of significant interest.

### 3.4 Investigation of electron transfer between TOH and AOX using Cyclic Voltammetry (CV)

An explanation for the observed synergy is the regeneration pathways between TOH and the tested AOX. To investigate this, CV assays were conducted to evaluate the ability of both curcumin and quercetin to regenerate TOH in aqueous solution to get closer to the emulsion model conditions.

#### 3.4.1 Cyclic Voltammetry (CV) of TOH at both neutral and acidic pH

**Fig. 7:**
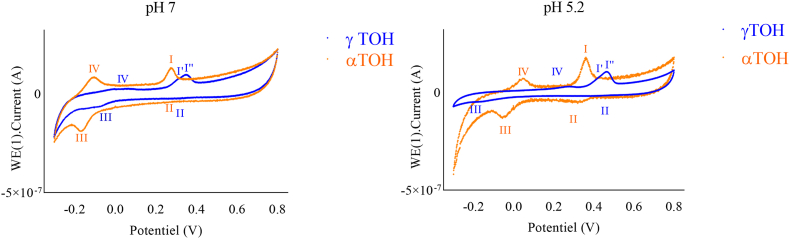
Cyclic voltammograms of TOH (10 μM) in NH_4_PF_6_ (0.1 mol.L^−1^) on a glassy carbon electrode at pH 7 and pH 5.2. Measurements were performed using a platinum wire counter electrode and an Ag/AgCl (3 M KCl) reference electrode. The scan rate was 0.25 V s^−1^.

**Fig. 8:**
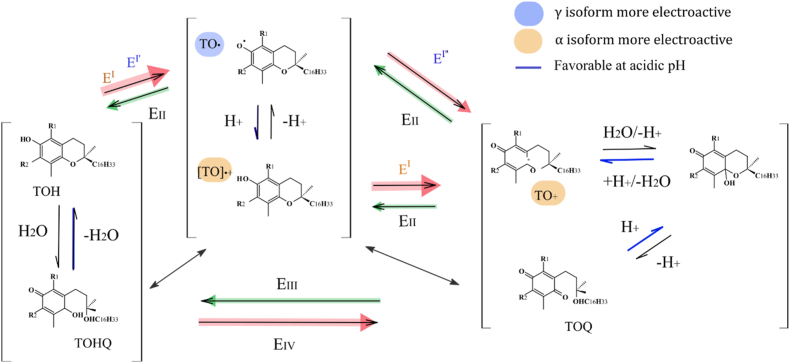
Proposed mechanism for the oxidation pathways of TOH as a function of isoform and pH.

The CV of TOH exhibited distinct behaviors under acidic and neutral pH conditions (Fig. 7), confirming that their oxidative behavior depends on the isoform, and is affected by the pH (Wilson et al., 2006). Based on these observations and previous literature, a proposed redox mechanism for αTOH and γTOH under both neutral and acidic pH is illustrated in Fig. 8. In the case of γTOH, the first oxidation peak (peak I, ∼0.3 V) appears as two distinct peaks, I′ and I″ with lower intensity. Additionally, two reduction peaks (II, 0.4 V and III, −0.17 V) are present at lower pH, whereas only one (III, −0.12 V) appeared at neutral conditions. For αTOH, peaks III (−0.32 V) and IV (−0.06 V) exhibit greater intensity than those of γTOH, suggesting that these reactions are less favorable. Peak I corresponds to a two-electron transfer via an electrochemical-chemical-electrochemical (ECE) mechanism, leading to the formation of TO^+^, a dienone cation (Giacomelli et al., 2004; Tan et al., 2011). The stability of this cation depends on the TOH isoform. According to Wilson et al. (2006) (Wilson et al., 2006), αTO^+^ is relatively stable in acetonitrile (CH_3_CN), whereas γTO^+^ is stable for only a few seconds. This instability likely explains why peaks III and IV are less intense for γTOH, as its oxidation products are less persistent and probably less detectable. Additionally, for γTOH, the CV features suggest two separate one-electron transfer steps (peak I and I’), likely due to the relative stability of the TO• radical intermediate, making it observable in the voltammogram. At neutral pH, this intermediate is rapidly hydrolyzed to tocopheryl quinone (TOQ). Under acidic conditions, hydrolysis is less favorable, which explains the appearance of reduction peak II. TOQ can subsequently undergo a chemical–electrochemical (CE) reaction, converting it into hydroquinone (TOHQ), which can be electrochemically reduced back to TOH.

#### 3.4.2 Cyclic Voltammetry (CV) of TOH at both neutral and acidic pH, in the presence of curcumin or quercetin

**Fig. 9:**
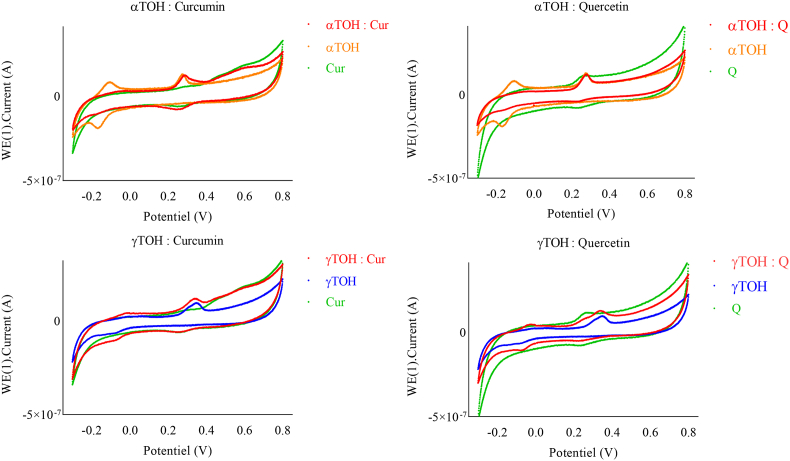
Cyclic voltammograms of TOH (10 μM), curcumin (Cur, 10 μM), quercetin (Q, 10 μM), and their equimolar combinations (TOH + Cur or TOH + Q) in NH_4_PF_6_ (0.1 mol L^−1^) on a glassy carbon electrode at pH 7. Measurements were performed using a platinum wire counter electrode and an Ag/AgCl (3 M KCl) reference electrode. The scan rate was 0.25 V s^−1^.

The CV of curcumin and quercetin showed no significant changes with pH (Fig. 9). For curcumin, two oxidation peaks (0.42 V and 0.65 V) and one reduction peak were observed at acidic pH, consistent with the findings of Stanić et al. (2008) (Stanić et al., 2008). However, at neutral pH, three oxidation peaks were observed (0.28 V; 0.46 V and 0.6 V). The related oxidation products may have the potential to reduce TOH, although further experiments are required to verify this.

For quercetin, only one oxidation peak was detected at the applied scan rate 0.39 V at acidic pH and 0.26 V at neutral pH. This peak may correspond to a two-electron transfer process, as previously reported by Timbola (Timbola et al., 2006). However, other studies conducted in buffered solutions have identified two distinct oxidation peaks (Sokolová et al., 2011, 2012). Such discrepancies are likely due to differences in experimental conditions, particularly scan rate or medium composition.

The observed effects of combining curcumin or quercetin with TOH were similar for both antioxidants but varied depending on the pH. At neutral pH, the second oxidation/reduction peaks were considerably diminished; however, this reduction was less pronounced for γTOH. The hypothesis that TOH regeneration underlies the observed synergistic effect appears more justified for αTOH than for γTOH, where the effect may also be influenced by partitioning phenomena. Fig. S2 in the supplementary data reveals a direct correlation between the decrease of the curcumin-related peak at (0.65 V) and the increase of the second redox peak of αTOH (at −0.06V). This observation highlights the role of curcumin in inhibiting secondary oxidation-reduction pathways.

**Fig. 10:**
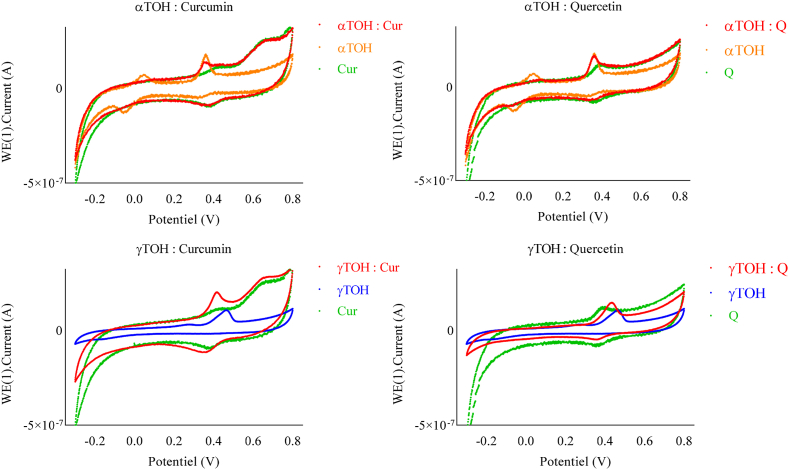
Cyclic voltammograms of TOH (10 μM), curcumin (Cur, 10 μM), quercetin (Q, 10 μM), and their equimolar combinations (TOH + Cur or TOH + Q) in NH_4_PF_6_ (0.1 mol L^−1^) on a glassy carbon electrode at pH 4. Measurements were performed using a platinum wire counter electrode and an Ag/AgCl (3 M KCl) reference electrode. The scan rate was 0.25 V s^−1^.

At acidic pH (Fig. 10), reduction is complete, and a reduction peak (at ∼ 0.3V) appears, even though it was missing in the absence of an antioxidant. This observation may be related to a larger amount of recovered TOH, resulting in sufficient TO^+^ to be reduced back to TOH. Such results nuance the conclusions drawn in the previous section and indicate that additional mechanisms beyond simple regeneration may modulate TOH recycling in the emulsion system. By linking kinetic parameters to molecular mechanisms, this approach moves beyond a simple global efficacy assessment, providing a mechanistically informed framework for tailoring antioxidant combinations. This integrated understanding opens the way to more stable PUFA-enriched formulations that meet both naturalness and performance requirements in the agri-food sector.

#### 3.4.3 Correlation between CV data and the WIM-CAT findings

CV assesses whether the oxidation products of TOH remain electrochemically accessible and thermodynamically reducible, a prerequisite for antioxidant regeneration, whereas the WIM-CAT approach captures the effective and kinetic impact of antioxidant mixtures on oxidative stability within structured interfacial nanoemulsions. In such systems, antioxidant performance is governed by molecular mobility, phase partitioning, radical propagation pathways, and steric and interfacial constraints. CV results indicate that TOH oxidation is strongly dependent on pH and isoform and that TOH regeneration in the presence of curcumin or quercetin is thermodynamically feasible. However, WIM-CAT reveals that this regenerative potential is effectively expressed mainly at low TOH concentrations. At higher TOH levels, accumulation of oxidation products likely hampers productive antioxidant interactions, either through direct reactions of tocopheroxyl radicals or TOH with lipid substrates and hydroperoxides, or through the involvement of quinone-type species in alternative oxidation pathways. In this context, WIM-CAT suggests that increasing the relative concentration of quercetin may partially restore antioxidant efficiency. Notably, γ-TOH consistently exhibits higher protection factors despite a weaker electrochemical regeneration signature, which can be rationalized by its preferential localization at the oil–water interface compared to α-TOH. This interfacial positioning enhances interactions with surface-active polyphenolic antioxidants during initiation and early propagation stages, while the greater stability of γ-TOH oxidation products likely provides a physical interaction advantage that compensates for reduced redox efficiency. CV further demonstrates that neutral pH promotes rapid hydrolysis of tocopheroxyl intermediates into tocopheryl quinone, whereas acidic conditions retard hydrolysis and allow electrochemical reduction back to TOH. Across all pH conditions, curcumin modulates the emergence of secondary redox processes for all TOH isoforms, while quercetin shows reduced effectiveness with α-TOH under acidic conditions but a more pronounced regenerative response with γ-TOH. In contrast, WIM-CAT reveals that curcumin exhibits diminished antioxidant efficacy under acidic conditions, whereas quercetin maintains strong functional interactions with tocopherols particularly γ-TOH although a pro-oxidant effect persists at elevated quercetin concentrations. While suppression of secondary oxidation peaks in CV confirms the thermodynamic feasibility of TOH regeneration, WIM-CAT demonstrates that additional mechanisms beyond redox thermodynamics govern antioxidant performance. The α and β kinetic descriptors derived from WIM-CAT show that secondary antioxidants can either decelerate (curcumin) or accelerate (quercetin) oxidation kinetics, reflecting shifts in dominant propagation pathways driven by the capacity of parent and/or oxidized antioxidant species to stabilize or destabilize lipid oxidation products such as hydroperoxides. Consequently, lag-phase–derived parameters from WIM-CAT only partially correlate with CV outcomes, whereas the inclusion of kinetic descriptors provides deeper insight into the evolving interactions between antioxidants and lipid oxidation products during propagation. Overall, these results demonstrate that antioxidant synergy in nanoemulsions arises from the combined effects of redox accessibility and interfacial positioning, but is ultimately governed by the physicochemical environment, matrix composition—including the intrinsic reactivity of the secondary antioxidant—and kinetic limitations, rather than molecular thermodynamics alone.

## 4 Conclusions

This study integrates the WIM-CAT kinetic framework with cyclic voltammetry to clarify how tocopherol–polyphenol combinations modulate lipid oxidation in oil-in-water nanoemulsions. The findings indicate that antioxidant synergy is not inherent to the molecules themselves, but rather emerges from the interaction between isoform-specific reactivity, polyphenol structure, concentration, and matrix conditions. γ-Tocopherol with curcumin and α-tocopherol with quercetin were the most effective pairs, particularly at low tocopherol levels, while higher tocopherol concentrations reduced synergy due to the onset of pro-oxidant behavior. Weibull modeling revealed distinct roles during the initiation and propagation phases, with curcumin providing sustained protection and quercetin generating oxidation products that can accelerate propagation at higher levels. Electrochemical data further investigated the chemical reactivity in between species depending on the medium conditions (pH) and suggest that synergy can involve tocopherol regeneration, especially for α-tocopherol. In contrast, the behavior of γ-tocopherol seems governed by the stability of its oxidized intermediates and its interfacial localization. Overall, this work highlights the importance of considering pH, metal ions, and antioxidant ratios factors commonly encountered during food processing when designing natural antioxidant systems. The combined WIM-CAT assay is a first approach to identify synergistic antioxidant combinations that offer a mechanistic and predictive tool to guide the formulation of more stable PUFA-rich emulsions, supporting the development of products with improved oxidative resistance and extended shelf life.

## Ethics statement

No ethics statement is required for the research presented in this study since neither procedures nor raw materials involved animals or animal-derived products.

## Credit author contributions

Camille Robichon: Data curation, Formal analysis, Investigation, Methodology, Software Writing – review and editing, Writing – original draft. Erwann Durand: Investigation, Supervision, Resources, Writing–review and editing. Jayaruwan G. Gamaethiralalage: Data curation, Formal analysis, Investigation, Methodology, Resources, Writing – review and editing. Philippe Bohuon: Investigation, Software, Formal analysis, Writing – review and editing. Nathalie Barouh: Investigation, Resources, Writing – review and editing. Bruno Baréa: Investigation, Resources, Writing – review and editing. Francis Courtois: Investigation, Writing – review and editing. Frédéric Fine: Funding acquisition. Louis C. P. M. de Smet: Resources, Writing – review and editing. Pierre Villeneuve: Investigation, Supervision, Resources, Writing–review and editing.

## Declaration of competing interest

The authors declare that they have no known competing financial interests or personal relationships that could have appeared to influence the work reported in this paper.

## Abbreviations

AE, antioxidant efficiency; αTOH, α-tocopherol; γTOH, γ-tocopherol; AOX, second antioxidants combined with tocopherols; CE, Chemical–Electrochemical mechanism; CF, Combination factor; CV, Cyclic voltammetry; DLS, Dynamic light scattering; ECE, Electrochemical–Chemical–Electrochemical mechanism; FAME, Fatty acid methyl esters; GC, Gas chromatography; HPLC, High-performance liquid chromatography; ICα/ICβ, confidence interval for α/β parameters; LagP, lag phase; PDF, probability density function; PI, polydispersity index; PUFA, polyunsaturated fatty acids; PV, peroxide value; RMSE, Root mean square error; T0.2, emulsion containing 0.2 μM of tocopherol; T0.6, emulsion containing 0.6 μM of tocopherol; TFA, total fatty acid; TOH, Tocopherols; TOHQ, Tocopheryl hydroquinone; TOQ, Tocopheryl quinone; WIM CAT, Weibull interaction modelling conjugated autoxidizable triene.
