# Supplementary material for: Decoding Tocopherol–Polyphenol interactions in oil-in-water emulsions through combined WIM-CAT and CV assays
Source: Curr Res Food Sci. 2026 Feb 9;12:101344. doi: 10.1016/j.crfs.2026.101344 (PMC12914691; doi:10.1016/j.crfs.2026.101344)
Supplement: Multimedia component 1 [file mmc1.docx]

Table S1 : Model parameters (α, β, Tlag) statistical significance and performance measures (R², RMSE)

|  | [TOH] | Ratio | α | stat p<0.05 | β | stat p<0.05 | Tlag (h) | stat p<0.05 | R² | RMSE |
| --- | --- | --- | --- | --- | --- | --- | --- | --- | --- | --- |
| αTOH : Cur pH 7 | 0.6 µM | (1:3) | 52.2 ± 0.2 | a | 3.7 ± 0.3 | a | 20.8 ± 1.9 | a | 0.94 | 0.11 |
|  | 0.6 µM | (1:1) | 33.6 ± 0.2 | b | 4.5 ± 0.4 | a | 13.5 ± 1.1 | b | 0.97 | 0.08 |
|  | 0.6 µM | (1:0.3) | 34.3 ± 0.2 | b | 4.6 ± 0.4 | a | 14.1 ± 1.2 | b | 0.97 | 0.08 |
|  | 0.2 µM | (1:3) | 32.9 ± 1.3 | a | 3.0 ± 0.4 | a | 7.9 ± 1.6 | a | 0.91 | 0.13 |
|  | 0.2 µM | (1:1) | 27.3 ± 1.0 | b | 3.0 ± 0.3 | a | 5.8 ± 1.3 | ab | 0.93 | 0.11 |
|  | 0.2 µM | (1:0.3) | 19.8 ± 0.6 | c | 2.7 ± 0.3 | a | 2.8 ± 0.7 | b | 0.95 | 0.08 |
| αTOH : Cur pH 7 (Fe II) | 0.6 µM | (1:3) | 45.0 ± 0.1 | a | 4.0 ± 0.3 | a | 17.7 ± 1.4 | a | 0.96 | 0.09 |
|  | 0.6 µM | (1:1) | 33.1 ± 0.1 | b | 4.0 ± 0.4 | a | 11.7 ± 1.4 | b | 0.96 | 0.09 |
|  | 0.6 µM | (1:0.3) | 29.1 ± 0.2 | c | 3.6 ± 0.3 | a | 8.7 ± 1.1 | b | 0.96 | 0.08 |
|  | 0.2 µM | (1:3) | 22.0 ± 0.7 | a | 3.9 ± 0.4 | a | 6.4 ± 0.8 | a | 0.96 | 0.07 |
|  | 0.2 µM | (1:1) | 19.2 ± 0.4 | b | 2.8 ± 0.3 | b | 2.7 ± 0.4 | b | 0.95 | 0.08 |
|  | 0.2 µM | (1:0.3) | 15.3 ± 0.6 | c | 1.7 ± 0.2 | c | 0.2 ± 0.2 | c | 0.92 | 0.09 |
| αTOH : Cur pH 4 | 0.6 µM | (1:3) | 41.1 ± 1.8 | a | 2.4 ± 0.3 | a | 7.3 ± 1.8 | a | 0.80 | 0.18 |
|  | 0.6 µM | (1:1) | 30.1 ± 0.9 | b | 2.7 ± 0.3 | a | 5.7 ± 1.4 | a | 0.92 | 0.12 |
|  | 0.6 µM | (1:0.3) | 33.4 ± 0.4 | c | 6.0 ± 0.6 | b | 15.0 ± 0.2 | b | 0.97 | 0.07 |
|  | 0.2 µM | (1:3) | 25.0 ± 0.5 | a | 3.6 ± 0.4 | a | 7.1 ± 1.1 | a | 0.96 | 0.09 |
|  | 0.2 µM | (1:1) | 25.7 ± 0.6 | a | 3.5 ± 0.4 | a | 6.9 ± 1.1 | a | 0.95 | 0.09 |
|  | 0.2 µM | (1:0.3) | 15.1 ± 0.4 | b | 3.5 ± 0.4 | b | 3.3 ± 0.7 | b | 0.97 | 0.08 |
| αTOH : Cur pH 4 (Fe II) | 0.6 µM | (1:3) | 43.6 ± 0.7 | a | 2.9 ± 0.2 | a | 11.3 ± 1.2 | a | 0.96 | 0.08 |
|  | 0.6 µM | (1:1) | 39.6 ± 0.3 | b | 4.1 ± 0.2 | b | 15.4 ± 0.6 | b | 0.99 | 0.04 |
|  | 0.6 µM | (1:0.3) | 31.6 ± 0.6 | c | 4.7 ± 0.5 | b | 12.8 ± 1.4 | ab | 0.95 | 0.1 |
|  | 0.2 µM | (1:3) | 19.7 ± 0.7 | a | 2.7 ± 0.4 | a | 2.8 ± 0.9 | a | 0.94 | 0.11 |
|  | 0.2 µM | (1:1) | 21.8 ± 0.4 | a | 3.7 ± 0.3 | ab | 5.0 ± 0.2 | b | 0.97 | 0.07 |
|  | 0.2 µM | (1:0.3) | 13.1 ± 0.4 | b | 3.7 ± 0.6 | b | 3.0 ± 0.8 | a | 0.96 | 0.09 |
| γTOH : Cur pH 7 | 0.6 µM | (1:3) | 96.9 ± 1.0 | a | 3.0 ± 1.0 | a | 18.2 ± 1.8 | a | 0.94 | 0.07 |
|  | 0.6 µM | (1:1) | 61.0 ± 1.3 | b | 3.6 ± 1.3 | a | 25.7 ± 2.6 | a | 0.90 | 0.14 |
|  | 0.6 µM | (1:0.3) | 42.2 ± 0.3 | c | 6.5 ± 0.3 | b | 23.3 ± 0.7 | a | 0.99 | 0.04 |
|  | 0.2 µM | (1:3) | 61.8 ± 0.8 | a | 4.0 ± 0.8 | a | 28.4 ± 1.6 | a | 0.96 | 0.08 |
|  | 0.2 µM | (1:1) | 40.2 ± 0.4 | b | 5.5 ± 0.4 | b | 19.8 ± 1.0 | b | 0.98 | 0.06 |
|  | 0.2 µM | (1:0.3) | 29.3 ± 0.3 | c | 4.5 ± 0.3 | ab | 11.6 ± 1.4 | c | 0.98 | 0.05 |
| γTOH : Cur pH 7 (Fe II) | 0.6 µM | (1:3) | 89.0 ± 2.0 | a | 2.9 ± 2.0 | a | 21.1 ± 3.5 | a | 0.81 | 0.15 |
|  | 0.6 µM | (1:1) | 55.0 ± 0.5 | b | 5.6 ± 0.5 | b | 20.1 ± 1.3 | a | 0.98 | 0.06 |
|  | 0.6 µM | (1:0.3) | 39.5 ± 0.8 | c | 3.3 ± 0.8 | a | 12.1 ± 1.5 | b | 0.94 | 0.1 |
|  | 0.2 µM | (1:3) | 50.3 ± 0.9 | a | 4.0 ± 0.9 | a | 21.1 ± 1.8 | a | 0.94 | 0.1 |
|  | 0.2 µM | (1:1) | 33.0 ± 0.6 | b | 4.1 ± 0.6 | a | 12.1 ± 1.2 | b | 0.96 | 0.09 |
|  | 0.2 µM | (1:0.3) | 23.3 ± 0.3 | c | 4.6 ± 0.3 | a | 8.5 ± 0.7 | b | 0.98 | 0.06 |
| γTOH : Cur pH 4 | 0.6 µM | (1:3) | 72.2 ± 1.8 | a | 2.4 ± 1.8 | a | 21.3 ± 3.4 | a | 0.83 | 0.13 |
|  | 0.6 µM | (1:1) | 41.7 ± 0.3 | b | 5.7 ± 0.3 | b | 21.3 ± 0.7 | a | 0.99 | 0.05 |
|  | 0.6 µM | (1:0.3) | 40.1 ± 0.7 | c | 4.7 ± 0.7 | b | 17.6 ± 1.6 | a | 0.95 | 0.1 |
|  | 0.2 µM | (1:3) | 32.6 ± 0.4 | a | 3.6 ± 0.4 | a | 10.4 ± 1.5 | a | 0.97 | 0.06 |
|  | 0.2 µM | (1:1) | 29.6 ± 0.2 | b | 4.8 ± 0.2 | b | 12.1 ± 0.6 | a | 0.99 | 0.04 |
|  | 0.2 µM | (1:0.3) | 24.8 ± 0.2 | c | 6.2 ± 0.2 | c | 11.8 ± 0.6 | a | 0.99 | 0.05 |
| γTOH : Cur pH 4 (Fe II) | 0.6 µM | (1:3) | 36.1 ± 0.6 | a | 3.1 ± 0.6 | a | 9.5 ± 1.1 | a | 0.97 | 0.07 |
|  | 0.6 µM | (1:1) | 38.8 ± 0.4 | b | 5.4 ± 0.4 | b | 18.7 ± 0.9 | b | 0.98 | 0.06 |
|  | 0.6 µM | (1:0.3) | 33.2 ± 0.6 | c | 4.6 ± 0.6 | b | 13.5 ± 1.2 | c | 0.96 | 0.09 |
|  | 0.2 µM | (1:3) | 30.1 ± 0.5 | a | 3.8 ± 0.5 | a | 9.6 ± 1.0 | a | 0.96 | 0.07 |
|  | 0.2 µM | (1:1) | 25.9 ± 0.2 | b | 4.7 ± 0.2 | a | 9.9 ± 0.5 | a | 0.99 | 0.04 |
|  | 0.2 µM | (1:0.3) | 21.7 ± 0.2 | c | 6.2 ± 0.2 | c | 10.1 ± 0.6 | a | 0.99 | 0.05 |
| αTOH : Q pH 7 (Fe II) | 0.6 µM | (1:3) | 42.2 ± 0.7 | a | 0.0 ± 0.0 | a | 20.7 ± 1.7 | a | 0.94 | 0.11 |
|  | 0.6 µM | (1:1) | 24.1 ± 0.4 | b | 0.0 ± 0.0 | a | 9.7 ± 1.0 | b | 0.96 | 0.08 |
|  | 0.6 µM | (1:0.3) | 20.9 ± 0.6 | c | 0.0 ± 0.0 | b | 3.4 ± 1.6 | c | 0.93 | 0.10 |
|  | 0.2 µM | (1:3) | 26.2 ± 0.5 | a | 0.0 ± 0.0 | a | 9.6 ± 1.1 | a | 0.95 | 0.09 |
|  | 0.2 µM | (1:1) | 18.9 ± 0.4 | b | 0.0 ± 0.0 | a | 5.4 ± 0.8 | b | 0.96 | 0.07 |
|  | 0.2 µM | (1:0.3) | 13.5 ± 0.6 | c | 0.0 ± 0.0 | b | 0.2 ± 0.2 | c | 0.92 | 0.09 |
| αTOH : Q pH 4 | 0.6 µM | (1:3) | 32.5 ± 0.2 | a | 0.0 ± 0.0 | a | 19.8 ± 0.6 | a | 0.99 | 0.04 |
|  | 0.6 µM | (1:1) | 31.4 ± 0.3 | b | 0.0 ± 0.0 | a | 19.4 ± 0.9 | a | 0.98 | 0.07 |
|  | 0.6 µM | (1:0.3) | 30.9 ± 0.6 | b | 0.0 ± 0.0 | b | 13.8 ± 1.5 | b | 0.95 | 0.11 |
|  | 0.2 µM | (1:3) | 23.6 ± 0.4 | a | 0.0 ± 0.0 | a | 11.7 ± 1.3 | a | 0.95 | 0.09 |
|  | 0.2 µM | (1:1) | 19.7 ± 1.0 | b | 0.0 ± 0.0 | b | 3.9 ± 1.3 | b | 0.87 | 0.17 |
|  | 0.2 µM | (1:0.3) | 14.5 ± 1.0 | c | 0.0 ± 0.0 | b | 1.0 ± 1.0 | b | 0.86 | 0.17 |
| αTOH : Q pH 4 (Fe II) | 0.6 µM | (1:3) | 38.6 ± 0.7 | a | 0.0 ± 0.0 | a | 17.7 ± 1.8 | a | 0.94 | 0.11 |
|  | 0.6 µM | (1:1) | 31.4 ± 0.8 | b | 0.0 ± 0.0 | a | 11.6 ± 1.4 | b | 0.92 | 0.13 |
|  | 0.6 µM | (1:0.3) | 28.2 ± 0.6 | c | 0.0 ± 0.0 | a | 10.6 ± 1.5 | b | 0.94 | 0.11 |
|  | 0.2 µM | (1:3) | 21.9 ± 0.6 | a | 0.0 ± 0.0 | a | 8.8 ± 1.7 | a | 0.92 | 0.13 |
|  | 0.2 µM | (1:1) | 17.1 ± 1.3 | b | 0.0 ± 0.0 | b | 1.4 ± 1.2 | b | 0.80 | 0.20 |
|  | 0.2 µM | (1:0.3) | 12.4 ± 0.8 | c | 0.0 ± 0.0 | b | 0.9 ± 0.9 | b | 0.90 | 0.14 |
| γTOH : Q pH 7 | 0.6 µM | (1:3) | 73.7 ± 0.3 | a | 0.0 ± 0.0 | a | 44.9 ± 1.9 | a | 0.99 | 0.05 |
|  | 0.6 µM | (1:1) | 45.9 ± 1.0 | b | 0.0 ± 0.0 | b | 18.1 ± 2.1 | b | 0.92 | 0.13 |
|  | 0.6 µM | (1:0.3) | 26.1 ± 0.5 | c | 0.0 ± 0.0 | b | 5.7 ± 0.9 | c | 0.95 | 0.08 |
|  | 0.2 µM | (1:3) | 35.5 ± 0.5 | a | 0.0 ± 0.0 | a | 15.4 ± 1.1 | a | 0.97 | 0.07 |
|  | 0.2 µM | (1:1) | 29.5 ± 0.2 | b | 0.0 ± 0.0 | a | 13.4 ± 0.6 | a | 0.99 | 0.04 |
|  | 0.2 µM | (1:0.3) | 26.2 ± 0.2 | c | 0.0 ± 0.0 | a | 10.7 ± 0.5 | b | 0.99 | 0.04 |
| γTOH : Q pH 7 (Fe II) | 0.6 µM | (1:3) | 64.3 ± 0.8 | a | 0.0 ± 0.0 | a | 39.9 ± 1.7 | a | 0.94 | 0.11 |
|  | 0.6 µM | (1:1) | 42.5 ± 0.2 | b | 0.0 ± 0.0 | b | 25.8 ± 0.6 | b | 0.99 | 0.04 |
|  | 0.6 µM | (1:0.3) | 25.9 ± 0.7 | c | 0.0 ± 0.0 | c | 4.8 ± 3.7 | c | 0.93 | 0.10 |
|  | 0.2 µM | (1:3) | 31.9 ± 0.5 | a | 0.0 ± 0.0 | a | 12.6 ± 1.1 | a | 0.97 | 0.08 |
|  | 0.2 µM | (1:1) | 23.0 ± 0.3 | b | 0.0 ± 0.0 | a | 8.2 ± 0.7 | b | 0.98 | 0.05 |
|  | 0.2 µM | (1:0.3) | 21.4 ± 0.4 | c | 0.0 ± 0.0 | b | 5.1 ± 0.7 | c | 0.97 | 0.07 |
| γTOH : Q pH 4 | 0.6 µM | (1:3) | 60.2 ± 0.5 | a | 0.0 ± 0.0 | a | 42.0 ± 1.1 | a | 0.97 | 0.08 |
|  | 0.6 µM | (1:1) | 32.2 ± 0.6 | b | 0.0 ± 0.0 | b | 13.4 ± 1.3 | b | 0.95 | 0.09 |
|  | 0.6 µM | (1:0.3) | 22.9 ± 0.6 | c | 0.0 ± 0.0 | c | 5.2 ± 1.0 | c | 0.94 | 0.09 |
|  | 0.2 µM | (1:3) | 29.6 ± 0.4 | a | 0.0 ± 0.0 | a | 13.9 ± 0.9 | a | 0.97 | 0.07 |
|  | 0.2 µM | (1:1) | 17.2 ± 0.6 | b | 0.0 ± 0.0 | b | 3.3 ± 1.0 | b | 0.93 | 0.11 |
|  | 0.2 µM | (1:0.3) | 18.5 ± 0.2 | b | 0.0 ± 0.0 | a | 7.2 ± 0.4 | c | 0.99 | 0.04 |
| γTOH : Q pH 4 (Fe II) | 0.6 µM | (1:3) | 46.3 ± 0.9 | a | 0.0 ± 0.0 | a | 19.8 ± 2.0 | a | 0.92 | 0.12 |
|  | 0.6 µM | (1:1) | 25.4 ± 0.3 | b | 0.0 ± 0.0 | b | 14.2 ± 0.9 | b | 0.97 | 0.07 |
|  | 0.6 µM | (1:0.3) | 18.8 ± 0.5 | c | 0.0 ± 0.0 | a | 4.5 ± 0.9 | c | 0.94 | 0.09 |
|  | 0.2 µM | (1:3) | 26.3 ± 0.5 | a | 0.0 ± 0.0 | a | 11.0 ± 1.3 | a | 0.95 | 0.10 |
|  | 0.2 µM | (1:1) | 18.2 ± 0.2 | b | 0.0 ± 0.0 | a | 7.4 ± 0.6 | b | 0.98 | 0.06 |
|  | 0.2 µM | (1:0.3) | 15.4 ± 0.2 | c | 0.0 ± 0.0 | a | 5.9 ± 0.5 | b | 0.99 | 0.05 |

Figure S2: 95 Cycles voltammograms of 10 µM antioxidant, TOH, and its equimolar combination with Cur in 0.1 mol·L⁻¹ NH₄PF₆ on a glassy carbon electrode at pH 7. Measurements were performed using a platinum wire counter electrode and an Ag/AgCl (3 M KCl) reference electrode. The scan rate was 0.25 V·s⁻¹.
